# On the Capacity Regions of Degraded Relay Broadcast Channels with and without Feedback

**DOI:** 10.3390/e22070784

**Published:** 2020-07-17

**Authors:** Bingbing Hu, Ke Wang, Yingying Ma, Youlong Wu

**Affiliations:** 1 School of Information Science and Technology, ShanghaiTech University, Shanghai 201210, China; hubb@shanghaitech.edu.cn (B.H.); wangke@shanghaitech.edu.cn (K.W.); mayy1@shanghaitech.edu.cn (Y.M.); 2 Shanghai Institute of Microsystem and Information Technology, Chinese Academy of Sciences, Shanghai 200050, China; 3 University of Chinese Academy of Sciences, Beijing 100049, China

**Keywords:** relay broadcast channel, capacity region, feedback

## Abstract

The four-node relay broadcast channel (RBC) is considered, in which a transmitter communicates with two receivers with the assistance of a relay node. We first investigate three types of physically degraded RBCs (PDRBCs) based on different degradation orders among the relay and the receivers’ observed signals. For the discrete memoryless (DM) case, only the capacity region of the second type of PDRBC is already known, while for the Gaussian case, only the capacity region of the first type of PDRBC is already known. In this paper, we step forward and make the following progress: (1) for the first type of DM-PDRBC, a new outer bound is established, which has the same rate expression as an existing inner bound, with only a slight difference on the input distributions; (2) for the second type of Gaussian PDRBC, the capacity region is established; (3) for the third type of PDRBC, the capacity regions are established both for DM and Gaussian cases. Besides, we also consider the RBC with relay feedback where the relay node can send the feedback signal to the transmitter. A new coding scheme based on a hybrid relay strategy and a layered Marton’s coding is proposed. It is shown that our scheme can strictly enlarge Behboodi and Piantanida’s rate region, which is tight for the second type of DM-PDRBC. Moreover, we show that capacity regions of the second and third types of PDRBCs are exactly the same as that without feedback, which means feedback cannot enlarge capacity regions for these types of RBCs.

## 1. Introduction

In the classical three-node relay channel [1,2], a relay node helps the communication between the transmitter and receiver. If the relay node also wants to decode a private message sent by the transmitter, then this channel turns out to be a partially cooperative relay broadcast channel (RBC) [3,4]. The capacity region of partially cooperative RBC is established for the case of degraded message sets [4], where the transmitter has a common message for both destinations and a private message for the relay. The fully cooperative RBC [3] is a more general model where both destinations can serve as the relay and receiver. A two-user degraded broadcast channel with conferencing and degraded message sets was studied in [5]. In [6], a degraded RBC was studied and an achievable rate region and distortion performance based on the decode-forward relay strategies proposed. In [7], the physically degraded and state-dependent RBC with cooperating decoders was investigated. A compress-forward inner bound was established for RBC with confidential messages in [8]. Partially and fully cooperative state-dependent relay broadcast channels with perfect causal channel state information (CSI) were considered in [9]. In [10], rate regions for two types of partially cooperative RBC with non-causal side information were established. The fully and partially cooperative RBCs with rate-limited feedback were studied in [11].

In many practical scenarios, receivers are unwilling to serve as relay nodes for other users due to the concern for privacy leakage or energy overuse. It is thus more practical to introduce exclusive relay nodes to assist the communication, leading to the so-called dedicated RBCs [12,13]. This model is particularly common in modern communication systems such as 4G or 5G cellular network, where relay nodes are incorporated into networks to improve the communication rate and enlarge the service range as well. The dedicated RBCs have been widely studied in the literature. In [14], the capacity region of a single-relay dedicated Gaussian RBC was established when one receiver’s observed signal was a degraded form of the other receiver’s, and the stronger receiver’s observed signal was a degraded form of the relay’s. In [15], an RBC with three parallel unmatched subchannels was studied, which was shown to enlarge the family of RBCs and relay channels for which the capacity region was known. For *r*-relay (r≤2) dedicated RBCs, the general outer bounds and capacity results for several classes of broadcast relay channels were investigated in [16], in which the capacity region of the so-called semi-degraded BC was established. The achievable rate region for *K*-receiver RBCs with one dedicated relay node was proposed in [17]. Multiple-input multiple-output (MIMO) RBC with direct links based on a weighted sum-rate criterion was studied in [18]. A new achievable rate region generalizing Marton’s coding to two-relay two-user RBCs was proposed in [19]. For multiple-relay RBC, single relay selection schemes using either selective digital relaying or selective analog relaying were proposed, which were shown to improve significantly the bit error probability performance [20]. Inner bounds were proposed for a single-relay dedicated RBC with fast fading and state feedback in [21].

**Figure 1:**
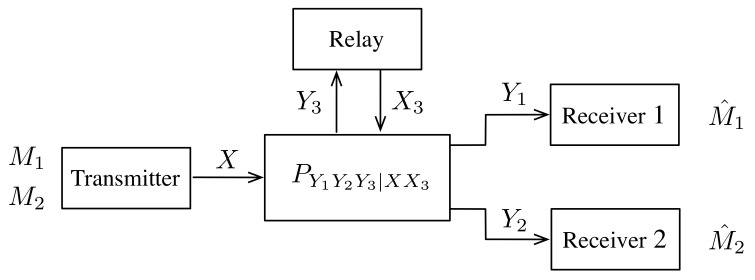
Relay broadcast channel.

In spite of the enormous amount of prior work on dedicated RBCs, the capacity region is still unknown. Moreover, it has been shown that feedback cannot enlarge the capacity region for some three-node degraded communication channels, such as degraded BCs and the degraded relay channel. Whether this result still holds for the four-node degraded RBCs is also an interesting problem. In this paper, we revisit the four-node dedicated RBC $p_{Y_{1}Y_{2}Y_{3}|XX_{3}}\left(y_{1},y_{2},y_{3}|x,x_{3}\right)$, as depicted in Figure 1, where *X* is a symbol sent by the transmitter, $Y_{3}$ and $Y_{k}$, for k∈{1,2}, are symbols observed by the relay and receiver *k*, respectively, and $X_{3}$ is a symbol created by observing $Y_{3}$ and to be sent by the relay. Even for the degraded case, the capacity region is still unknown, except for some special cases. The main challenges to establish its capacity region mainly come from the following three parts:

- Various definitions of degraded RBCs: Due to the five channel parameters $\left(X,X_{3},Y_{1},Y_{2},Y_{3}\right)$, there could be multiple definitions of degraded RBCs, which poses difficulties finding a unified single-letter capacity region for all types of degraded RBCs. For example, in [16], Behboodi and Piantanida defined RBC as degraded/semi-degraded if it satisfied one of the following conditions.
  - Condition 1: $X-\left(X_{3},Y_{3}\right)-\left(Y_{1},Y_{2}\right)$ and $\left(X,X_{3}\right)-Y_{1}-Y_{2}$.
  - Condition 2: $X-\left(X_{3},Y_{3}\right)-Y_{2}$ and $X-\left(X_{3},Y_{1}\right)-Y_{3}$.
  In [16], the RBC satisfying Condition 1 was called degraded RBC and the RBC satisfying Condition 2 semi-degraded degraded RBC. In Condition 1, the relay’s observed signal is “stronger” than both receivers’. In Condition 2, the relay’s observed signal is “stronger” than one receiver’s, but “weaker” than the other receiver’s. Apart from these conditions, one can also define new types of degraded RBCs by changing the location of $X_{3}$ in the Markov chains or assuming that the relay’s observed signal is “weaker” than both receivers’, e.g., satisfying:
  - Condition 3: $X-\left(X_{3},Y_{1}\right)-Y_{2}-Y_{3}$.
  Notice that for the discrete memoryless (DM) degraded RBCs satisfying the three conditions above, only the capacity region of RBC satisfying Condition 2 is already known, while for the Gaussian case, only the capacity region of RBC satisfying Condition 1 is already known [14].
- Achievability proof: To establish the capacity regions of different types of degraded RBCs, an ideal solution is to find a general inner bound and outer bound that match for all types of degraded RBCs. Since the capacity results for the degraded broadcast channel (BC) and degraded relay channel are already known, one may come up with a mixed scheme by combining the optimal schemes used in these channels. Unfortunately, this mixed scheme turns out to be not always optimal [13,14]. An alternative way is to exploit the degradation structure and carefully the design coding scheme for each specific type, such as the work done in [14,16]. Note that this is not an easy work, either. For example, the capacity region of the degraded RBC that satisfies the aforementioned Condition 1 is still unknown, except for the Gaussian case.
- Converse proof: The cut-set bound is strictly suboptimal, since it is not even tight for the degraded BCs. An outer bound for the degraded BCs satisfying the aforementioned Condition 1 or Condition 2 was established in [16]. It is not known whether this outer bound is optimal, as this outer bound does not match with the proposed inner bound in the given rate expressions. Moreover, the outer bound could be invalid for other types of degraded RBCs. Thus, it would be necessary to build outer bounds for different types of degrade RBCs and prove their tightness.

Now, we summarize the main contributions of this paper as follows:

- For DM-physically degraded RBCs (PDRBCs) satisfying Condition 1, a new outer bound is established, which has the same rate expression as an existing inner bound, with only a slight difference on the input distributions; for Gaussian PDRBCs satisfying Condition 2, the capacity region is established based on entropy power inequality (EPI) [22] and an appropriate relay strategy; for PDRBCs satisfying Condition 3, the capacity regions are established both for the DM and Gaussian cases.
- We propose a new coding scheme for general RBC with relay feedback where the relay node can send the feedback signal to the transmitter. The new scheme is based on a hybrid relay strategy and a layered Marton’s coding. It is shown that our scheme can strictly enlarge Behboodi and Piantanida’s rate region, which is tight for the second type of DM-PDRBC. Moreover, we show that capacity regions of the second and third types of PDRBCs are exactly the same as that without feedback, which means that feedback cannot enlarge capacity regions for these types of RBCs.

The rest of the paper is organized as follows. The system model is introduced in Section 2. Various definitions of degraded RBCs are given in Section 3. In Section 4, we state our main capacity results for DM and Gaussian PDRBC. The result on RBC with feedback is give in Section 5, and its proof is given in Section 6. The proof of the outer bounds on the capacity regions of DM-PDRBC with feedback are stated in Section 7. The proof of capacity regions for Gaussian PDRBC is presented in Section 8. Section 9 concludes this paper.

Notation: We use capital letters to denote random variables and small letters for their realizations, e.g., *X* and *x*. Define a function $C\left(x\right):=\frac{1}{2}\log_{2}\left(1+x\right)$. For nonnegative integers k,j, let $X_{k}^{j}:=\left(X_{k,1},\ldots ,X_{k,j}\right)$. Given a random variable *X*, we write its probability mass function (pmf) $p_{X}\left(x\right)$ as p(x) for short. For an integer *N*, we use [1:N] to denote {1,…,N}. Given a real value α with 0≤α≤1, let $\bar{\alpha }:=1-\alpha$. Considering *K* sequences $X_{1}^{n},\ldots ,X_{K}^{n}$ each of *n*-length, the joint typical set $T_{\epsilon }^{\left(n\right)}\left(p(x_{1},x_{2},\ldots ,x_{K})\right)$ denotes the set of all sequences ($X_{1}^{n},\ldots ,X_{K}^{n}$) when they are join typical, where the parameter ϵ is a positive value involved in the definition of joint typicality and tends to zero as the length *n* goes to infinity. More details and properties about $T_{\epsilon }^{\left(n\right)}\left(p(x_{1},x_{2},\ldots x_{K})\right)$ can be found in [22] (p. 29).

## 2. System Model

Consider a four-node discrete memoryless relay broadcast channel (DM-RBC) in which there is one transmitter, two receivers, and one relay that helps the transmitter to communicate with the receivers, as depicted in Figure 1. This channel consists of five finite alphabets ($X,X_{3},Y_{1},Y_{2},Y_{3}$) and a collection of pmf $p(y_{1},y_{2},y_{3}|x,x_{3})$ on $Y_{1}\times Y_{2}\times Y_{3}$, one for each $X\times X_{3}$. Here, x∈X is a symbol sent by the transmitter, $y_{3}\in Y_{3}$ and $y_{k}\in Y_{k}$, for k∈{1,2}, are symbols observed by the relay and receiver *k*, respectively, and $x_{3}\in X_{3}$ is a symbol created by observing $y_{3}$ and will be sent by the relay.

The transmitter wishes to communicate a message $M_{k}\in \left[1:2^{nR_{k}}\right]$ to receiver *k*, for k∈{1,2}, with the assistance of a relay node, where *n* denotes the block length of transmission. In the following subsections, we introduce the DM-RBC without and with feedback, respectively.

### 2.1. RBC without Feedback

A $\left(2^{nR_{1}},2^{nR_{2}},n\right)$-code for DM-RBC has:

- two message sets $M_{1}=\left[1:2^{nR_{1}}\right]$ and $M_{2}=\left[1:2^{nR_{2}}\right]$,
- a source encoder that maps messages $\left(M_{1},M_{2}\right)$ to the channel input $X_{i}\left(M_{1},M_{2}\right)$, for each time i∈[1:n],
- a relay encoder that maps $Y_{3}^{i-1}$ to a sequence $X_{3,i}\left(Y_{3}^{i-1}\right)$, where $Y_{3}^{i-1}:=\left(Y_{3,1},\ldots ,Y_{3,i-1}\right)$, for time i∈[1:n],
- two decoders that estimate ${\hat{M}}_{1}$ and ${\hat{M}}_{2}$ based on $Y_{1}^{n}$ and $Y_{2}^{n}$, respectively, where $Y_{k}^{n}:=\left(Y_{k,1},\ldots ,Y_{k,n}\right)$, for k=1,2.

Suppose $M_{k}$ is uniformly distributed over the message set $M_{k}$. A rate region $\left(R_{1},R_{2}\right)$ is called achievable if for every blocklength *n*, there exists a $\left(2^{nR_{1}},2^{nR_{2}},n\right)$-code such that the average probability of error:

$$P_{e}^{\left(n\right)}=\mathrm{Pr}\left[{\hat{M}}_{1}\neq M_{1}\;\mathrm{or}\;{\hat{M}}_{2}\neq M_{2}\right]$$

tends to zero as *n* tends to infinity. The capacity region $C_{\mathrm{NoFb}}$ is the closure of the set of all achievable rate pairs $\left(R_{1},R_{2}\right)$.

### 2.2. RBC with Feedback

Consider a DM-RBC with feedback from the relay and users to the transmitter. A $\left(2^{nR_{1}},2^{nR_{2}},n\right)$-code for this channel has:

- two message sets $M_{1}=\left[1:2^{nR_{1}}\right]$ and $M_{2}=\left[1:2^{nR_{2}}\right]$,
- a source encoder that maps messages $\left(M_{1},M_{2},Y_{1}^{i-1},Y_{2}^{i-1},Y_{3}^{i-1}\right)$ to the channel input $X_{i}\left(M_{1},M_{2}\right)$, for each time i∈[1:n],
- a relay encoder that maps $Y_{3}^{i-1}$ to a sequence $X_{3,i}\left(Y_{3}^{i-1}\right)$, for i∈[1:n],
- two decoders that estimate ${\hat{M}}_{1}$ and ${\hat{M}}_{2}$ based on $Y_{1}^{n}$ and $Y_{2}^{n}$, respectively.

Suppose $M_{k}$ is uniformly distributed over the message set $M_{k}$. A rate region $\left(R_{1},R_{2}\right)$ is called achievable if for every blocklength *n*, there exists a $\left(2^{nR_{1}},2^{nR_{2}},n\right)$-code such that the average probability of error:

$$P_{e}^{\left(n\right)}=\mathrm{Pr}\left[{\hat{M}}_{1}\neq M_{1}\;\mathrm{or}\;{\hat{M}}_{2}\neq M_{2}\right]$$

tends to zero as *n* tends to infinity. The capacity region $C_{\mathrm{Fb}}$ is the closure of the set of all achievable rate pairs $\left(R_{1},R_{2}\right)$.

## 3. Definitions of Various PDRBCs

In this paper, we mainly focus on the PDRBC. Let $C_{\mathrm{PD}}$ denote the capacity region of PDRBC. Without loss of generality, assume $Y_{2}$ is a random degradation of $Y_{1}$, which implies that output $Y_{1}$ is “stronger” than $Y_{2}$. According to the degradation order among $Y_{1},Y_{2}$ and $Y_{3}$, we consider three types of PDRBC.

**Definition** **1.** *An RBC $p(y_{1},y_{2},y_{3}|x,x_{3})$ is said to be*

- *Type-I PDRBC if $X-\left(X_{3},Y_{3}\right)-\left(Y_{1},Y_{2}\right)$ and $\left(X,X_{3}\right)-Y_{1}-Y_{2}$ form Markov chains.*
- *Type-II PDRBC if $X-\left(X_{3},Y_{3}\right)-Y_{2}$ and $X-\left(X_{3},Y_{1}\right)-Y_{3}$ form Markov chains.*
- *Type-III PDRBC if $X-\left(X_{3},Y_{1}\right)-Y_{2}-Y_{3}$ forms a Markov chain.*

Type-I PDRBC and Type-II PDRBC are not new, which were introduced by [16]. In Type-I DM-PDRBC, the relay observes a “stronger” symbol than both receivers, and its capacity region is still unknown, except for the Gaussian case [14]. In Type-II PDRBC, the relay observes a “stronger” signal than one receiver, but a “weaker” signal than another receiver. For the DM case, the capacity region of Type-II PDRBC was established in [16] (Theorem 7), which is the set of rate pairs $\left(R_{1},R_{2}\right)$ such that:

(1a) $$\begin{array}{ccc} R_{2} & \leq & \min\left\{I\left(U,X_{3};Y_{2}\right),I\left(U;Y_{3}|X_{3}\right)\right\}, \end{array}$$

(1b) $$\begin{array}{ccc} R_{1}+R_{2} & \leq & \min\left\{I\left(U,X_{3};Y_{2}\right),I\left(U;Y_{3}|X_{3}\right)\right\}+I\left(X;Y_{1}|U,X_{3}\right), \end{array}$$

for some pmf $p\left(u,x_{3}\right)p\left(x|u\right)$. Type-III PDRBC is newly defined, in which the relay’s observed signal is “weaker” than both receivers’. Type-II and Type-III PDRBCs could characterize some practical communication scenarios when some receivers are closer to the transmitter and thus have a better received signal than relay nodes.

More generally, stochastically degraded RBC is defined as below.

**Definition** **2.** *An RBC $p(y_{1},y_{2},y_{3}|x,x_{3})$ is said to be:*

- *Type-I stochastically degraded RBC if there exist random variables $\left({\tilde{Y}}_{1},{\tilde{Y}}_{3}\right)$ such that $p\left({\tilde{y}}_{1}|x,x_{3}\right)=p_{Y_{1}|XX_{3}}\left({\tilde{y}}_{1}|x,x_{3}\right)$ and $p\left({\tilde{y}}_{3}|x,x_{3}\right)=p_{Y_{3}|XX_{3}}\left({\tilde{y}}_{3}|x,x_{3}\right)$ and $X-\left(X_{3},{\tilde{Y}}_{3}\right)-\left({\tilde{Y}}_{1},Y_{2}\right)$ and $\left(X,X_{3}\right)-{\tilde{Y}}_{1}-Y_{2}$ form Markov chains.*
- *Type-II stochastically degraded RBC if there exist random variables $\left({\tilde{Y}}_{1},{\tilde{Y}}_{3}\right)$ such that $p\left({\tilde{y}}_{1}|x,x_{3}\right)=p_{Y_{1}|XX_{3}}\left({\tilde{y}}_{1}|x,x_{3}\right)$ and $p\left({\tilde{y}}_{3}|x,x_{3}\right)=p_{Y_{3}|XX_{3}}\left({\tilde{y}}_{3}|x,x_{3}\right)$ and $X-\left(X_{3},{\tilde{Y}}_{3}\right)-Y_{2}$ and $X-\left(X_{3},{\tilde{Y}}_{1}\right)-{\tilde{Y}}_{3}$ form Markov chains.*
- *Type-III stochastically degraded RBC if there exist random variables $\left({\tilde{Y}}_{1},{\tilde{Y}}_{2}\right)$ such that $p\left({\tilde{y}}_{1}|x,x_{3}\right)=p_{Y_{1}|XX_{3}}\left({\tilde{y}}_{1}|x,x_{3}\right)$ and $p\left({\tilde{y}}_{2}|x,x_{3}\right)=p_{Y_{2}|XX_{3}}\left({\tilde{y}}_{3}|x,x_{3}\right)$ and $X-\left(X_{3},{\tilde{Y}}_{1}\right)-{\tilde{Y}}_{2}-Y_{3}$ forms a Markov chain.*

**Remark** **1.** *Following the similar proof in [14] (Lemma 1), when $p\left(y_{1},y_{2},y_{3}|x,x_{3}\right)=p\left(y_{3}|x,x_{3}\right)p\left(y_{1},y_{2}|y_{3},x_{3}\right)$, the capacity region of RBC depends only on the marginal conditional distributions $p(y_{1}|x_{3},y_{3})$ and $p(y_{2}|x_{3},y_{3})$. Thus, the capacity region of Type-I PDRBC holds when it is stochastically degraded.*

### Gaussian PDRBCs

Consider the Gaussian RBC, which can be described as:

$$\begin{array}{ccc} Y_{k} & = & X+X_{3}+Z_{k},\;k=1,2,\\ Y_{3} & = & X+Z_{3}, \end{array}$$

where $Z_{1},Z_{2}$, and $Z_{3}$ are Gaussian noise components with zero mean and variances $\sigma_{1}^{2},\sigma_{2}^{2}$, and $\sigma_{3}^{2}$, respectively. Assume average transmission power constraint *P* on the transmitter and $P_{r}$ on the relay.

Similar to the discrete memoryless case, Gaussian PDRBC can be divided into three types according to the degradation order among the outputs received at the receivers and relay:

- Type-I Gaussian PDRBC: The channel outputs $\left(Y_{1},Y_{2},Y_{3}\right)$ are equivalent to:
  $$\begin{array}{ccc} Y_{3} & = & X+Z_{3},\\ Y_{1} & = & X+X_{3}+Z_{3}+{\hat{Z}}_{a},\\ Y_{2} & = & X+X_{3}+Z_{3}+{\hat{Z}}_{a}+{\tilde{Z}}_{a}, \end{array}$$
  where ${\hat{Z}}_{a}∼N\left(0,\sigma_{1}^{2}-\sigma_{3}^{2}\right)$ and ${\tilde{Z}}_{a}∼N\left(0,\sigma_{2}^{2}-\sigma_{1}^{2}\right)$ are independent. Note that in this case, $X-\left(X_{3},Y_{3}\right)-\left(Y_{1},Y_{2}\right)$ and $\left(X,X_{3}\right)-Y_{1}-Y_{2}$ form Markov chains, and the noise variances satisfy:
  (2) $$\sigma_{2}^{2}\geq \sigma_{1}^{2}\geq \sigma_{3}^{2}.$$
- Type-II Gaussian PDRBC: The channel outputs $\left(Y_{1},Y_{2},Y_{3}\right)$ are equivalent to:
  $$\begin{array}{ccc} Y_{1} & = & X+X_{3}+Z_{1},\\ Y_{3} & = & X+Z_{1}+{\hat{Z}}_{b},\\ Y_{2} & = & X+X_{3}+Z_{1}+{\hat{Z}}_{b}+{\tilde{Z}}_{b}, \end{array}$$
  where ${\hat{Z}}_{b}∼N\left(0,\sigma_{3}^{2}-\sigma_{1}^{2}\right)$ and ${\tilde{Z}}_{b}∼N\left(0,\sigma_{2}^{2}-\sigma_{3}^{2}\right)$ are independent. Note that in this case, $X-\left(X_{3},Y_{3}\right)-Y_{2}$ and $X-\left(X_{3},Y_{1}\right)-Y_{3}$ form Markov chains, and the noise variances satisfy:
  (3) $$\sigma_{2}^{2}\geq \sigma_{3}^{2}\geq \sigma_{1}^{2}.$$
- Type-III Gaussian PDRBC: The channel outputs $\left(Y_{1},Y_{2},Y_{3}\right)$ are equivalent to:
  $$\begin{array}{ccc} Y_{1} & = & X+X_{3}+Z_{1},\\ Y_{2} & = & X+X_{3}+Z_{1}+{\hat{Z}}_{c},\\ Y_{3} & = & X+Z_{1}+{\hat{Z}}_{c}+{\tilde{Z}}_{c}, \end{array}$$
  where ${\hat{Z}}_{c}∼N\left(0,\sigma_{2}^{2}-\sigma_{1}^{2}\right)$ and ${\tilde{Z}}_{c}∼N\left(0,\sigma_{3}^{2}-\sigma_{2}^{2}\right)$ are independent. Note that in this case, $X-\left(X_{3},Y_{1}\right)-Y_{2}-Y_{3}$ forms a Markov chain, and the noise variances satisfy:
  (4) $$\sigma_{3}^{2}\geq \sigma_{2}^{2}\geq \sigma_{1}^{2}.$$

**Remark** **2.** *For Type-I Gaussian PDRBC, the capacity region was established in [14], which is the set of rate pairs $\left(R_{1},R_{2}\right)$ such that:*

(5a) $$\begin{array}{c} R_{1}\leq \min\left\{C\left(\bar{\alpha }\theta \frac{P}{\sigma_{3}^{2}}\right),C\left(\frac{\theta P+\theta_{r}P_{r}+2\sqrt{\alpha \theta \theta_{r}PP_{r}}}{\sigma_{1}^{2}}\right)\right\}, \end{array}$$

(5b) $$\begin{array}{c} R_{2}\leq C\left(\frac{\bar{\theta }P+{\bar{\theta }}_{r}P_{r}+2\sqrt{\beta \bar{\theta }{\bar{\theta }}_{r}PP_{r}}}{\theta P+\theta_{r}P_{r}+2\sqrt{\alpha \theta \theta_{r}PP_{r}}+\sigma_{2}^{2}}\right),\; \end{array}$$

(5c) $$\begin{array}{c} R_{1}+R_{2}\leq C\left(\frac{\bar{\beta }\bar{\theta }P+\bar{\alpha }\theta P}{\sigma_{3}^{2}}\right), \end{array}$$

*where $0\leq \alpha ,\beta ,\theta ,\theta_{r}\leq 1$. The capacity regions of Type-II and Type-III Gaussian PDRBC are still unknown.*

## 4. Capacity Results for PDRBC without Feedback

### 4.1. Discrete Memoryless PDRBC

**Theorem** **1.** *For Type-I PDRBC, the inner bound on the capacity region consists of all rate pairs $\left(R_{1},R_{2}\right)$ such that:*

(6a) $$\begin{array}{ccc} R_{2} & \leq & I(U,V;Y_{2}), \end{array}$$

(6b) $$\begin{array}{ccc} R_{1}+R_{2} & \leq & I\left(U,V;Y_{2}\right)+I\left(X;Y_{3}|U,V,X_{3}\right),\; \end{array}$$

(6c) $$\begin{array}{ccc} R_{1}+R_{2} & \leq & I\left(U,V;Y_{2}\right)+I\left(X,X_{3};Y_{1}|U,V\right), \end{array}$$

(6d) $$\begin{array}{ccc} R_{1}+R_{2} & \leq & I(X;Y_{3}|V,X_{3}), \end{array}$$

*for some pmf $p\left(u,v\right)p\left(x_{3}|v\right)p\left(x|u,x_{3}\right)$. The outer bound on the capacity region has the same rate constraints as (6), but under the pmf $p\left(u,v\right)p\left(x_{3}|u\right)p\left(x|u,x_{3}\right)$ and H(V|U)=0.*

*For Type-III PDRBC, the capacity region is the set of rate pairs $\left(R_{1},R_{2}\right)$ such that:*

(7a) $$\begin{array}{ccc} R_{1} & \leq & I(X;Y_{1}|U,X_{3}=x_{3}), \end{array}$$

(7b) $$\begin{array}{ccc} R_{2} & \leq & I(U;Y_{2}|X_{3}=x_{3}), \end{array}$$

*for some value $x_{3}\in X_{3}$ and pmf p(u,x).*

**Proof.**  For Type-I PDRBC, the inner bound is obtained by letting $\left(V,X_{3},U\right)$ satisfy the Markov chain $V-X_{3}-U$ in the inner bound presented in [16] (Theorem 9), and the outer bound is proven in Section 7.1. For Type-III PDRBC, the outer bound is given in Section 7.3, and the achievable scheme is to let the relay send a constant value $x_{3}$ and the transmitter use the traditional superposition coding to send the source messages, i.e., the weak receiver’s intended message is stored in a cloud center codeword $u^{n}$ and will be decoded by both receivers, and the strong receiver’s intended message is conveyed through a satellite codeword $x^{n}$, which will be decoded by the strong receiver. □

### 4.2. Gaussian PDRBC

**Theorem** **2.** *For Type-II Gaussian PDRBC, the capacity region is the set of rate pairs $\left(R_{1},R_{2}\right)$ such that:*

(8a) $$\begin{array}{ccc} R_{1} & \leq & C\left(\frac{\alpha P}{\sigma_{1}^{2}}\right), \end{array}$$

(8b) $$\begin{array}{ccc} R_{2} & \leq & \min\left\{C\left(\frac{\bar{\alpha }P+P_{r}}{\alpha P+\sigma_{2}^{2}}\right),C\left(\frac{(\beta -\alpha )P}{\alpha P+\sigma_{3}^{2}}\right)\right\},\; \end{array}$$

*where 0≤α,β≤1, and β≥α.*

*For Type-III Gaussian PDRBC, the capacity region is the set of rate pairs $\left(R_{1},R_{2}\right)$ such that:*

(9) $$\begin{array}{ccc} R_{1} & \leq & C\left(\frac{\alpha P}{\sigma_{1}^{2}}\right),\;R_{2}\leq C\left(\frac{\bar{\alpha }P}{\alpha P+\sigma_{2}^{2}}\right), \end{array}$$

*where 0≤α≤1.*

**Proof.**  See the proof in Section 8.1 and Section 8.2. □

**Figure 2:**
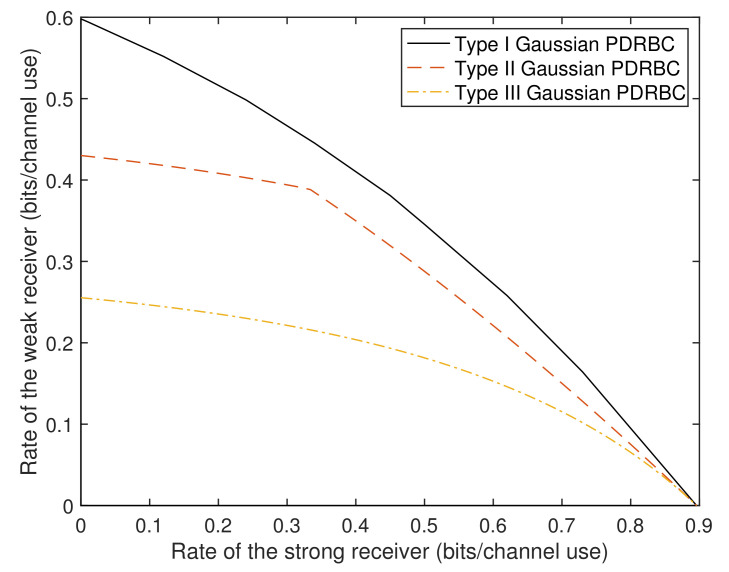
Capacity regions of three types of Gaussian physically degraded relay broadcast channel (PDRBC) when P=5, $P_{r}=10$, $\sigma_{\pi (1)}^{2}=1$, $\sigma_{\pi (2)}^{2}=2.5$, and $\sigma_{\pi (3)}^{2}=11$.

In Figure 2, we plot numerical results on the capacity regions for the three types of PDRBC. The chosen channel parameters are the same as those in [14], i.e., P=5, $P_{r}=10$, $\sigma_{\pi (1)}^{2}=1$, $\sigma_{\pi (2)}^{2}=2.5$, and $\sigma_{\pi (3)}^{2}=11$, where π(·) is some permutation on {1,2,3} such that the conditions in (2), (3), and (4) are satisfied for the corresponding types of PDRBCs. From this figure, we can see that Type-I Gaussian PDRBC has the largest capacity region, while Type-III Gaussian PDRBC has the smallest. This is reasonable because in Type-I, the relay observes the best signal and thus can help the receivers’ decoding to the largest extent, while in Type-III, the relay observes the weakest signal and thus cannot offer much help on receivers’ decoding as Type-I and Type-II PDRBCs. We observe that the boundary of the capacity region for Type-III PDRBC is not smooth in the middle, and this is mainly due to the existing minimum term in (8b).

## 5. Results on RBC with Feedback

**Theorem** **3.** *For the four-node RBC with feedback from the relay to the transmitter, the capacity region includes the set of rate pairs $\left(R_{1},R_{2}\right)$ such that:*

(10a) $$\begin{array}{ccc} R_{1} & \leq & I_{A}+I_{C}-I\left(U_{0};X_{3}|V\right), \end{array}$$

(10b) $$\begin{array}{ccc} R_{2} & \leq & I\left(V,U_{0},U_{2};Y_{2}\right)-I\left({\hat{Y}}_{3};Y_{3}|U_{0},U_{2},V,Y_{2}\right)-I\left(U_{2};X_{3}|U_{0},V\right), \end{array}$$

(10c) $$\begin{array}{ccc} R_{1}+R_{2} & \leq & I_{A}+I\left(U_{2};{\hat{Y}}_{3},Y_{2}|U_{0},V\right)-I\left(U_{2};U_{1},X_{3}|U_{0},V\right), \end{array}$$

(10d) $$\begin{array}{ccc} R_{1}+R_{2} & \leq & I_{B}+I\left(U_{0},U_{2},V;Y_{2}\right)-I\left({\hat{Y}}_{3};Y_{3}|U_{0},U_{2},V,Y_{2}\right)-I\left(U_{2};U_{1},X_{3}|U_{0},V\right), \end{array}$$

*for some pmf $p\left(v,u_{0},u_{1},u_{2},u_{3}\right)P\left({\hat{y}}_{3}|y_{3},u_{0}\right)$ and a function $x=f(v,u_{0},u_{1},u_{2},u_{3})$, where:*

$$\begin{array}{ccc} I_{A} & = & \min\{I\left(U_{0},V;Y_{1}\right)+I\left(X_{3};U_{0},Y_{1}|V\right)+I\left(U_{1};Y_{1}|U_{0},V_{0},X_{3}\right)-I\left({\hat{Y}}_{3};Y_{3}|U_{0},U_{1},V,X_{3},Y_{1}\right), \end{array}$$

(10e) $$\begin{array}{c} \;I\left(U_{0};X_{3},Y_{3}|V\right)+I\left(U_{1};Y_{3}|U_{0},V,X_{3}\right)\}, \end{array}$$

(10f) $$\begin{array}{ccc} I_{B} & = & \min\{I\left(U_{1};Y_{3}|U_{0},V,X_{3}\right),I\left(X_{3};U_{0},Y_{1},{\hat{Y}}_{3}|V\right)+I\left(U_{1};{\hat{Y}}_{3},Y_{1}|U_{0},V,X_{3}\right)\}, \end{array}$$

(10g) $$\begin{array}{ccc} I_{C} & = & I\left(U_{3};{\hat{Y}}_{3},Y_{1}|U_{0},U_{1},V,X_{3}\right)-I\left(U_{3};U_{2}|U_{0},V,U_{1},X_{3}\right). \end{array}$$

**Proof.**  In our scheme, source message $M_{1}$ is split into three independent sub-messages $\left(M_{1,c},M_{1,p1},M_{1,p2}\right)$, and $M_{2}$ is split into two independent sub-messages $\left(M_{2,c},M_{2,p}\right)$. Here, $M_{1,c}\in \left[1:2^{nR_{1,c}}\right]$ and $M_{2,c}\in \left[1:2^{nR_{2,c}}\right]$ are sub-messages that can be considered as a common part of the source messages since in our scheme, they will be decoded by both the relay and two receivers, $M_{1,p1}\in \left[1:2^{nR_{1,p1}}\right]$ is the sub-message that will be decoded by the relay and Receiver 1, $M_{1,p2}\in \left[1:2^{nR_{1,p2}}\right]$ is the sub-message that will be solely decoded by Receiver 1, and $M_{2,p}\in \left[1:2^{nR_{2,p}}\right]$ is the sub-message that will be solely decoded by Receiver 2. Hence, $R_{1}=R_{1,c}+R_{1,p1}++R_{1,p2}$ and $R_{2}=R_{2,c}+R_{2,p}$. The transmitter uses a layered Marton’s coding in which $\left(M_{1,c},M_{2,c}\right)$ is stored in the cloud center $U_{0}$ and will be decoded by the relay and both receivers, $M_{1,p1}$ is contained in $U_{1}$, a satellite of $U_{0}$, and will be decoded by the relay and Receiver 1, $M_{1,p2}$ is contained in $U_{3}$, a satellite of $U_{1}$, and will be solely decoded by Receiver 1, and $M_{2,p}$ is contained in $U_{2}$, a satellite of $U_{0}$, and will be solely decoded by Receiver 2. Apart from decoding $\left(M_{1,c},M_{2,c}\right)$ and $M_{1,p1}$, the relay also performs compress-forward to compress its observed symbol $Y_{3}$ as ${\hat{Y}}_{3}$ and sends the compression index back to the transmitter. The feedback message is sent by the transmitter in a cloud center *V* and will be decoded by both receivers. Each receiver decodes this compression message, reconstructs ${\hat{Y}}_{3}$, and then, uses it as side information to assist the decoding of source message. See the details in Section 6. □

**Remark** **3.** *Note that by exchanging the index “1” and “2” in *(10)*, the corresponding rate region is still achievable. This new rate region can be achieved by a similar scheme presented in Section 6, with simply exchanging the indices of Receiver 1 and Receiver 2. The union of both regions provides a potentially larger achievable rate region.*

Our scheme can strictly enlarge Behboodi and Piantanida’s rate regions in [16]. This can be seen by the following example.

**Example** **1.** *Consider a special case when $R_{2}=0$. In this case, the four-node RBC is equivalent to the classical three-node relay channel, where Receiver 2 can be ignored since it does not decode or send anything. Now, consider the Gaussian RBC with perfect feedback from the relay to the transmitter. The channel outputs are:*

$$\begin{array}{ccc} Y_{3} & = & g_{1}X+Z_{3},\\ Y_{1} & = & g_{2}X+g_{3}X_{3}+Z_{2}, \end{array}$$

*where $g_{1}$, $g_{2}$, and $g_{3}$ are channel gains, $E|X^{2}|\leq P$ and $E|X_{3}^{2}|\leq P_{r}$, and $Z_{1}∼N\left(0,1\right)$ and $Z_{2}∼N\left(0,1\right)$ are independent Gaussian noises.*

*For this channel, it can be easily checked that Behboodi and Piantanida’s scheme in [16] reduces to either the decode-forward or compress-forward relay strategy. In *(10)*, by setting $U_{1},U_{2}$ to be constant, $V_{0}=U$, $V=X_{3}$, and $U_{3}=X$, our achievable rate region in Theorem 3 turns out to be:*

(11) $$R_{1}\leq \min\left\{I\left(X,X_{3};Y_{1}\right)-I\left({\hat{Y}}_{3};Y_{3}|U,X,X_{3},Y_{1}\right),I\left(U;Y_{3}|X_{3}\right)+I\left(X;{\hat{Y}}_{3},Y_{1}|U,X_{3}\right)\right\}.$$

*In our previous paper [11], we showed that when P=5, $P_{r}=1$, $g_{1}=1/d$, $g_{2}=1$, and $g_{3}=1/\left|1-d\right|$, for d=0.73,0.74,0.75,0.76, the rate *(11)* is strictly larger than the rate achieved by the decode-forward and compress-forward relay strategies.*

In [14], it was shown that for Type-I PDRBC with feedback from the users to the relay and transmitter, feedback cannot enlarge the capacity region. We obtain similar results for Type-II and Type-III PDRBCs as follows:

**Theorem** **4.** *For Type-II PDRBC and Type-III PDRBC with feedback from the relay and users to the transmitter, the capacity regions $C_{\mathrm{Fb}}$ are exactly the same as those without feedback, which means that feedback cannot enlarge the capacity regions for these types of RBCs.*

**Proof.**  The achievable rate regions are the same as the rate regions of the non-feedback case. The converse proof is given in Section 8. □

## 6. Coding Schemes for RBC with Feedback

We present a scheme based on block-Markov coding [22] that consists of B+1 blocks, where messages $M_{1,b}\in \left[1:2^{nR_{1}}\right]$ and $M_{2,b}\in \left[1:2^{nR_{2}}\right]$, for b∈[1:B], are sent to the receivers over B+1 blocks. Split message $M_{1,b}$ into $\left(M_{1,c,b},M_{1,p1,b},M_{1,p2,b}\right)$, where $M_{1,c,b}\in \left[1:2^{nR_{1,c}}\right]$ and $M_{1,pi,b}\in \left[1:2^{nR_{1,pi}}\right]$, for i=1,2, are independent of each other. Split message $M_{2,b}$ into $\left(M_{2,c,b},M_{2,p,b}\right)$, where $M_{2,c,b}\in \left[1:2^{nR_{2,c}}\right]$ and $M_{2,p,b}\in \left[1:2^{nR_{2,p}}\right]$, are independent of each other. Define:

$$M_{c,b}≜\left(M_{1,c,b},M_{2,c,b}\right),\;R_{c}≜R_{1,c}+R_{2,c}.$$

Let messages $M_{1,B+1}=M_{2,B+1}=1$, and assume $\left(M_{1,B+1},M_{2,B+1}\right)$ are known by the relay and receivers before communication. Note that the transmission takes place over B+1 blocks, and the messages $\left(M_{1,B+1},M_{2,B+1}\right)$ are deterministic; thus, the transmission rates of messages $M_{1}$ and $M_{2}$ are in fact equal to $R_{1}\frac{B}{B+1}$ and $R_{2}\frac{B}{B+1}$, respectively. In the limit n→∞ and B→∞, $R_{1}$ and $R_{2}$ approach these transmission rates. Therefore, we neglect this technicality in the following.

### 6.1. Codebook

Fix the pmf $p_{VU_{0}U_{1}U_{2}U_{3}}\left(v,u_{0},u_{1},u_{2},u_{3}\right)P_{{\hat{Y}}_{3}|Y_{3}U_{0}}\left({\hat{y}}_{3}|y_{3},u_{0}\right)$ and a function $x=f(v,u_{0},u_{1},u_{2},u_{3})$, where *V* is an auxiliary random variable (RV) in a cloud center and plays two roles: building dependence between the transmitter’s and relay’s sending symbols and containing the feedback message and the common part of the source message of previous block $M_{c,b-1}$; $U_{0}$ represents the satellite of *V* that contains $M_{c,b}$ and will be decoded by the relay and two receivers; $U_{1}$ represents the satellite of $U_{0}$ that contains sub-message $M_{1,p1,b}$ to be decoded by the relay and Receiver 1; $U_{2}$ represents the satellite of $U_{0}$ that contains sub-message $M_{2,p,b}$ to be solely decoded by Receiver 2; $U_{3}$ represents the satellite of $U_{1}$ that contains sub-message $M_{1,p2,b}$ to be solely decoded by Receiver 1; ${\hat{Y}}_{3}$ represents the satellite of *V* containing the compression version of the relay’s observed symbol $Y_{3}$. Here, RVs $\left(V,U_{0},U_{1},U_{2},U_{3}\right)$ are related to the transmitter’s encoding and $\left(V,{\hat{Y}}_{3}\right)$ are related to the the relay’s encoding.

For each block b∈[1:B+1], randomly and independently generate $2^{n(R_{c}+{\hat{R}}_{3})}$ sequences $v_{b}^{n}\left(m_{c,b-1},l_{b-1}\right)∼\prod_{i=1}^{n}p_{V}\left(v_{b,i}\right)$, with $l_{b-1}\in \left[1:2^{n{\hat{R}}_{3}}\right]$. For each $\left(m_{c,b-1},l_{b-1}\right)$, randomly and independently generate $2^{nR_{c}}$ sequences $u_{0,b}^{n}\left(m_{c,b}|m_{c,b-1},l_{b-1}\right)∼\prod_{i=1}^{n}p_{U_{0}|V}\left(u_{0,b,i}|v_{b,i}\right)$. For each $\left(m_{c,b-1},l_{b-1}\right)$, randomly and independently generate $2^{n(R_{1,p1}+{\hat{R}}_{1})}$ sequences $x_{3,b}^{n}\left(m_{1,p1,b-1},j_{1,b-1}|m_{c,b-1},l_{b-1}\right)∼\prod_{i=1}^{n}p_{X_{3}|V}\left(x_{3,b,i}|v_{b,i}\right)$, with $j_{1,b-1}\in \left[1:2^{n{\hat{R}}_{1}}\right]$. For each $\left(m_{c,b},m_{c,b-1},l_{b-1}\right)$, randomly and independently generate $2^{n(R_{1,p1}+{\hat{R}}_{1})}$ sequences $u_{1,b}^{n}\left(m_{1,p1,b},j_{1,b}|m_{c,b},m_{c,b-1},l_{b-1}\right)∼\prod_{i=1}^{n}p_{U_{1}|VU_{0}}\left(u_{1,b,i}|u_{0,b,i},v_{b,i}\right)$. For each $\left(m_{c,b},m_{c,b-1},l_{b-1}\right)$, randomly and independently generate $2^{n(R_{2,p}+{\hat{R}}_{2})}$ sequences $u_{2,b}^{n}\left(m_{2,p,b},j_{2,b}|m_{c,b},m_{c,b-1},l_{b-1}\right)∼\prod_{i=1}^{n}p_{U_{2}|VU_{0}}\left(u_{2,b,i}|u_{0,b,i},v_{b,i}\right)$, with $j_{2,b}\in \left[1:2^{n{\hat{R}}_{2}}\right]$. For each $\left(m_{1,p1,b},m_{1,p1,b-1},j_{1,b},m_{c,b},m_{c,b-1},l_{b-1}\right)$, randomly and independently generate $2^{n(R_{1,p2}+{\hat{\hat{R}}}_{1})}$ sequences $u_{3,b}^{n}\left(m_{1,p2,b},j_{3,b}|m_{1,p1,b},m_{1,p1,b-1},j_{1,b},m_{c,b},m_{c,b-1},l_{b-1}\right)∼\prod_{i=1}^{n}p_{U_{3}|U_{0}U_{1}VX_{3}}\left(u_{3,b,i}|u_{0,b,i},u_{1,b,i},v_{b,i},x_{3,b,i}\right)$, with $j_{3,b}\in \left[1:2^{n{\hat{\hat{R}}}_{1}}\right]$. For each $\left(m_{c,b-1},l_{b-1}\right)$, randomly and independently generate $2^{n{\hat{R}}_{3}}$ sequences ${\hat{y}}_{3,b}^{n}\left(l_{b}|m_{c,b-1},l_{b-1}\right)∼\prod_{i=1}^{n}p_{{\hat{Y}}_{3}|V}\left({\hat{y}}_{3,b,i}|v_{b,i}\right)$.

### 6.2. Transmitter’s Encoding

The transmitter performs a layered Marton’s coding. More specifically, in each block b∈[1:B+1], the transmitter looks for a message $j_{1,b-1}$ such that:

$$\left(u_{0,b}^{n},v_{b}^{n},x_{3,b}^{n}\right)\in T_{\epsilon }^{n}(p\left(u_{0},v,x_{3}\right)).$$

Then, it looks for a pair of message $\left(j_{1,b},j_{2,b}\right)$ such that:

$$\left(u_{0,b}^{n},u_{1,b}^{n},u_{2,b}^{n},v_{b}^{n},x_{3,b}^{n}\right)\in T_{\epsilon }^{n}(p\left(u_{0},u_{1},u_{2},v,x_{3}\right)).$$

By the covering lemma [22], $\left(j_{1,b-1},j_{1,b},j_{2,b}\right)$ can be found with high probability if:

(12a) $$\begin{array}{ccc} {\hat{R}}_{1} & \geq & I\left(U_{0};X_{3}|V\right)+\delta \left(\epsilon \right), \end{array}$$

(12b) $$\begin{array}{ccc} {\hat{R}}_{2} & \geq & I\left(U_{2};X_{3}|U_{0},V\right)+\delta \left(\epsilon \right), \end{array}$$

(12c) $$\begin{array}{ccc} {\hat{R}}_{1}+R_{2} & \geq & I\left(U_{2};U_{1},X_{3}|U_{0},V\right)+\delta \left(\epsilon \right). \end{array}$$

In the next step, the transmitter looks for $j_{3,b}$ such that:

$$\left(u_{0,b}^{n},u_{1,b}^{n},u_{2,b}^{n},u_{3,b}^{n},x_{3,b}^{n},v_{b}^{n}\right)\in T_{\epsilon }^{n}(p\left(u_{0},u_{1},u_{2},x_{3},v\right)).$$

By the covering lemma [22], $j_{3,b-1}$ can be found with high probability if:

(13) $${\hat{\hat{R}}}_{1}\geq I\left(U_{3};U_{2}|U_{0},U_{1},X_{3},V\right)+\delta \left(\epsilon \right).$$

Finally, the transmitter sends the channel input $x_{b}^{n}=f\left(v^{n},u_{0}^{n},u_{1,b}^{n},u_{2,b}^{n},u_{3,b}^{n}\right).$

### 6.3. Relay’s Encoding

The relay performs a hybrid strategy combining partial decode-forward and compress-forward. More specifically, in each block b∈[1:B+1], the relay first looks for a tuple of messages $\left({\hat{m}}_{c,b}^{\left(3\right)},{\hat{m}}_{1,p1,b}^{\left(3\right)},j_{1,b}^{\left(3\right)}\right)$ such that:

(14) $$\begin{array}{c} (u_{0,b}^{n}\left({\hat{m}}_{c,b}^{\left(3\right)}|m_{c,b-1},l_{b-1}\right),\;u_{1,b}^{n}\left({\hat{m}}_{1,p1,b}^{\left(3\right)},j_{1,b}^{\left(3\right)}|{\hat{m}}_{c,b}^{\left(3\right)},m_{c,b-1},l_{b-1}\right),\;v_{b}^{n}\left(m_{c,b-1},l_{b-1}\right),\\ \;\;\;x_{3,b}^{n}\left(m_{1,p1,b-1},j_{1,b-1}|m_{c,b-1},l_{b-1}\right),\;y_{3,b}^{n})\in T_{\epsilon }^{n}\left(p\left(u_{0},u_{1},v,x_{3},y_{3}\right)\right). \end{array}$$

Notice that $l_{b-1}$ is the message generated by the relay (which can be seen soon) and thus is already known; $m_{c,b-1}$ and $j_{1,b-1}$ can be obtained from the decoding of the previous block. By the packing lemma [22], the decoding is successful with high probability if:

(15a) $$\begin{array}{ccc} R_{1,p1}+{\hat{R}}_{1} & \leq & I\left(U_{1};Y_{3}|U_{0},V,X_{3}\right)-\delta \left(\epsilon \right), \end{array}$$

(15b) $$\begin{array}{ccc} R_{c}+R_{1,p1}+{\hat{R}}_{1} & \leq & I\left(U_{0};X_{3},Y_{3}|V\right)+I\left(U_{1};Y_{3}|U_{0},V,X_{3}\right)-\delta \left(\epsilon \right). \end{array}$$

Then, it compresses $y_{3,b}^{n}$ by looking for $l_{b}$ such that:

$$\begin{array}{c} \left(v_{b}^{n},\;y_{3,b}^{n},\;{\hat{y}}_{3,b}^{n}\right)\in T_{\epsilon }^{n}\left(p\left(v,y_{3},{\hat{y}}_{3}\right)\right). \end{array}$$

By the covering lemma, it is successful with high probability if:

(16) $${\hat{R}}_{3}\geq I\left({\hat{Y}}_{3};Y_{3}|V\right)+\delta \left(\epsilon \right).$$

A the end of block *b*, the relay forwards $l_{b}$ to the transmitter through the feedback link. If the feedback link’s capacity is sufficiently large such the capacity is larger than the rate of $l_{b}$, then $l_{b}$ can be perfectly known by the transmitter.

After decoding $\left(m_{c,b},l_{b},m_{1,p1,b},j_{1,b}\right)$, the relay sends $x_{3,b+1}^{n}\left(m_{1,p1,b},j_{1,b}|m_{c,b},l_{b}\right)$ in block b+1.

### 6.4. Decoding

Receiver 2 applies backward decoding to decode $\left(v_{b}^{n},u_{0,b}^{n},u_{2,b}^{n},{\hat{y}}_{3,b}^{n}\right)$, for b∈[1:B]. Specifically, after (B+1)-block transmission, assuming Receiver 2 has already successfully decoded $\left(m_{c,b},l_{b}\right)$ based on $y_{2,b+1}^{n}$, it looks for $\left({\hat{m}}_{c,b-1}^{\left(2\right)},{\hat{l}}_{b-1}^{\left(2\right)},{\hat{m}}_{2,p,b},{\hat{j}}_{2,b}\right)$ such that:

$$\begin{array}{c} (v_{b}^{n}\left({\hat{m}}_{c,b-1}^{\left(2\right)},{\hat{l}}_{b-1}^{\left(2\right)}\right),u_{0,b}^{n}\left(m_{c,b}|{\hat{m}}_{c,b-1}^{\left(2\right)},{\hat{l}}_{b-1}^{\left(2\right)}\right),\\ \;u_{2,b}^{n}\left({\hat{m}}_{2,p,b},{\hat{j}}_{2,b}|m_{c,b},{\hat{m}}_{c,b-1}^{\left(2\right)}{\hat{l}}_{b-1}^{\left(2\right)}\right),{\hat{y}}_{3,b}^{n}\left(l_{b}|{\hat{m}}_{c,b-1}^{\left(2\right)},{\hat{l}}_{b-1}^{\left(2\right)}\right),y_{2,b}^{n})\in T_{\epsilon }^{n}\left(p\left(v,u_{0},u_{2},{\hat{y}}_{3},y_{2}\right)\right). \end{array}$$

By the packing lemma and the induction on backward decoding, the decoding is successful with high probability if:

(17a) $$\begin{array}{ccc} R_{2,p}+{\hat{R}}_{2} & \leq & I\left(U_{2};{\hat{Y}}_{3},Y_{2}|U_{0},V\right)-\delta \left(\epsilon \right), \end{array}$$

(17b) $$\begin{array}{ccc} R_{c}+{\hat{R}}_{3}+R_{2,p}+{\hat{R}}_{2} & \leq & I\left(U_{0},U_{2},V;Y_{2}\right)+I\left({\hat{Y}}_{3};U_{0},U_{2},Y_{2}|V\right)-\delta \left(\epsilon \right). \end{array}$$

Receiver 1 applies backward decoding to decode $\left(v_{b}^{n},u_{0,b}^{n},u_{1,b}^{n},u_{3,b}^{n},{\hat{y}}_{3,b}^{n}\right)$, for b∈[1:B+1]. More specifically, after (B+1)-block transmission, assuming Receiver 1 has already successfully decoded $\left(m_{c,b},l_{b}\right)$ based on $y_{1,b+1}^{n}$, it first looks for a tuple of messages $\left({\hat{m}}_{c,b-1}^{\left(1\right)},{\hat{l}}_{b-1}^{\left(1\right)},{\hat{m}}_{1,p1,b}^{\left(1\right)},{\hat{j}}_{1,b}^{\left(1\right)}\right)$ such that:

$$\begin{array}{c} (v_{b}^{n}\left({\hat{m}}_{c,b-1}^{\left(1\right)},{\hat{l}}_{b-1}^{\left(1\right)}\right),u_{0,b}^{n}\left(m_{c,b}|{\hat{m}}_{c,b-1}^{\left(1\right)},{\hat{l}}_{b-1}^{\left(1\right)}\right),u_{1,b}^{n}\left({\hat{m}}_{1,p1,b}^{\left(1\right)},{\hat{j}}_{1,b}^{\left(1\right)}|m_{c,b},{\hat{m}}_{c,b-1}^{\left(1\right)},{\hat{l}}_{b-1}^{\left(1\right)}\right),\\ \;{\hat{x}}_{3,b}^{n}\left({\hat{m}}_{c,b-1}^{\left(1\right)},{\hat{l}}_{b-1}^{\left(1\right)}\right),{\hat{y}}_{3,b}^{n}\left(l_{b}|{\hat{m}}_{c,b-1}^{\left(1\right)},{\hat{l}}_{b-1}^{\left(1\right)}\right),y_{2,b}^{n})\in T_{\epsilon }^{n}\left(p\left(v,u_{0},u_{1},x_{3},{\hat{y}}_{3},y_{1}\right)\right). \end{array}$$

By the packing lemma and the induction on backward decoding, the decoding is successful with high probability if:

(18a) $$\begin{array}{ccc} R_{1,p1}+{\hat{R}}_{1} & \leq & I\left(X_{3};{\hat{Y}}_{3},Y_{1},U_{0}|V\right)+I\left(U_{1};{\hat{Y}}_{3},Y_{1}|U_{0},V,X_{3}\right)-\delta \left(\epsilon \right),\\ R_{c}+{\hat{R}}_{3}+R_{1,p1}+{\hat{R}}_{1} & \leq & I\left(U_{0},V;Y_{1}\right)+I\left(X_{3};Y_{1},U_{0}|V\right)+I\left(U_{1};Y_{1}|U_{0},V,X_{3}\right), \end{array}$$

(18b) $$\begin{array}{c} +I\left({\hat{Y}}_{3};U_{0},U_{1},X_{3},Y_{1}|V\right)-\delta \left(\epsilon \right). \end{array}$$

After successfully decoding $\left(m_{c,b-1},l_{b-1},m_{1,p1,b},j_{1,b}\right)$, Receiver 1 continues decoding $u_{3,b}^{n}$ by looking for $\left({\hat{m}}_{1,p2,b},{\hat{j}}_{3,b}\right)$ such that:

$$\begin{array}{c} (u_{3,b}^{n}\left({\hat{m}}_{1,p2,b},{\hat{j}}_{3,b}|m_{1,p1,b},m_{1,p1,b-1},j_{1,b},m_{c,b},m_{c,b-1},l_{b-1}\right),\\ \;v_{b}^{n},u_{0,b}^{n},u_{1,b}^{n},x_{3,b}^{n},{\hat{y}}_{3,b}^{n},y_{1,b}^{n})\in T_{\epsilon }^{n}\left(p\left(v,u_{0},u_{1},u_{3},x_{3},{\hat{y}}_{3},y_{1}\right)\right).\;\;\;\; \end{array}$$

By the packing lemma and the induction on backward decoding, the decoding is successful with high probability if:

(19) $$R_{1,p2}+{\hat{\hat{R}}}_{1}\leq I\left(U_{3};Y_{1},{\hat{Y}}_{3}|U_{0},U_{1},V,X_{3}\right)-\delta \left(\epsilon \right).$$

Using Fourier–Motzkin elimination [22] to eliminate $R_{1,p1},R_{1,p2},R_{2,p},R_{c},{\hat{R}}_{1},{\hat{R}}_{2},{\hat{R}}_{3}$, and ${\hat{\hat{R}}}_{1}$ and since δ(ϵ) tends to zero when *n* goes to *∞*, we obtain the inner bound in Theorem 3.

## 7. Outer Bounds for PDRBC with Feedback

In this section, we present outer bounds for the three types of PDRBC with feedback. Note that these outer bounds are also valid for cases without feedback.

### 7.1. Outer Bound for Type-I PDRBC with Feedback

Define:

(20) $$\begin{array}{c} U_{i}=\left(M_{2},Y_{1}^{i-1},Y_{2}^{i-1}\right),V_{i}=\left(Y_{1}^{i-1},Y_{2}^{i-1}\right).\; \end{array}$$

Introduce a time-sharing random variable *Q* that is uniformly distributed over [1:n] and independent of $\left(M_{1},M_{2},U^{n},V^{n},X^{n},X_{3}^{n},Y_{1}^{n},Y_{2}^{n},Y_{3}^{n}\right)$.

By Fano’s inequality, we have:

(21) $$\begin{array}{ccc} nR_{1} & \leq & I\left(M_{1};Y_{1}^{n},Y_{2}^{n}|M_{2}\right)+n\epsilon_{n}\\ & \overset{\left(a\right)}{=} & \sum_{i=1}^{n}I\left(M_{1},Y_{1}^{i-1},Y_{2}^{i-1};Y_{1,i},Y_{2,i}|U_{i},V_{i}\right)+n\epsilon_{n}\\ & \overset{\left(b\right)}{\leq } & \sum_{i=1}^{n}I\left(X_{i},X_{3,i},M_{1};Y_{1,i},Y_{2,i}|U_{i},V_{i}\right)+n\epsilon_{n}\\ & \overset{\left(c\right)}{=} & \sum_{i=1}^{n}I\left(X_{i},X_{3,i};Y_{1,i},Y_{2,i}|U_{i},V_{i}\right)+n\epsilon_{n}\\ & = & \sum_{i=1}^{n}I\left(X_{i},X_{3,i};Y_{1,i},Y_{2,i}|U_{i},V_{i},Q=i\right)+n\epsilon_{n}\\ & = & nI\left(X_{Q},X_{3,Q};Y_{1,Q},Y_{2,Q}|U_{Q},V_{Q},Q\right)+n\epsilon_{n}, \end{array}$$

where $\epsilon_{n}$ is a positive value that tends to zero as n→∞ by Fano’s inequality; (a) holds by the definition of $U_{i},V_{i}$; (b) follows because $X_{i}$ is a function of $\left(M_{1},M_{2},Y_{1}^{i-1},Y_{2}^{i-1},Y_{3}^{i-1}\right)$; (c) follows from Markov chain $\left(U_{i},V_{i},M_{1}\right)-\left(X_{i},X_{3,i}\right)-\left(Y_{1,i},Y_{2,i}\right)$. Here, the auxiliary random variable *Q* is not part of the channel variables and represents a convex combination of all rate points obtained in the absence of *Q*.

Similarly,

(22) $$\begin{array}{c} n(R_{1}+R_{2})\\ \leq I\left(M_{1},M_{2};Y_{1}^{n},Y_{2}^{n},Y_{3}^{n}\right)+n\epsilon_{n}\\ \overset{\left(a\right)}{=}\sum_{i=1}^{n}I\left(M_{1},M_{2};Y_{1,i},Y_{2,i},Y_{3,i}|V_{i},Y_{3}^{i-1}\right)+n\epsilon_{n}\\ \overset{\left(b\right)}{=}H\left(Y_{1,i},Y_{2,i},Y_{3,i}|V_{i},X_{3,i},Y_{3}^{i-1}\right)\\ \;-H\left(Y_{1,i},Y_{2,i},Y_{3,i}|X_{i},X_{3,i},V_{i}\right)+n\epsilon_{n}\\ \leq \sum_{i=1}^{n}I\left(X_{i};Y_{1,i},Y_{2,i},Y_{3,i}|X_{3,i},V_{i}\right)+n\epsilon_{n}\\ \overset{\left(c\right)}{=}\sum_{i=1}^{n}I\left(X_{i};Y_{3,i}|X_{3,i},V_{i}\right)+n\epsilon_{n}\\ =nI\left(X_{Q};Y_{3,Q}|X_{3,Q},V_{Q},Q\right)+n\epsilon_{n}, \end{array}$$

where (a) follows by the definition of $V_{i}$; (b) holds because $X_{3,i}$ is a function of $Y_{3}^{i-1}$, $X_{i}$ is a function of $\left(M_{1},M_{2},Y_{1}^{i-1},Y_{2}^{i-1},Y_{3}^{i-1}\right)$, and $\left(M_{1},M_{2},V_{i},Y_{3}^{i-1}\right)-\left(X_{i},X_{3,i}\right)-\left(Y_{1,i},Y_{2,i},Y_{3,i}\right)$ forms a Markov chain; (c) follows from the property of Type-I PDRBC, which has Markov chain $X_{i}-\left(X_{3,i},Y_{3,i},V_{i}\right)-\left(Y_{1,i},Y_{2,i}\right)$.

In addition,

(23) $$\begin{array}{ccc} nR_{2} & \leq & I\left(M_{2};Y_{2}^{n}\right)+n\epsilon_{n}\\ & = & \sum_{i=1}^{n}I\left(M_{2};Y_{2,i}|Y_{2}^{i-1}\right)+n\epsilon_{n}\\ & \leq & \sum_{i=1}^{n}I\left(M_{2},Y_{2}^{i-1};Y_{2,i}\right)+n\epsilon_{n}\\ & \leq & \sum_{i=1}^{n}I\left(M_{2},Y_{2}^{i-1},Y_{1}^{i-1};Y_{2,i}\right)+n\epsilon_{n}\\ & = & \sum_{i=1}^{n}I\left(U_{i},V_{i};Y_{2,i}\right)+n\epsilon_{n}\\ & = & nI\left(U_{Q},V_{Q};Y_{2,Q}|Q\right)+n\epsilon_{n}\\ & \leq & nI\left(U_{Q},V_{Q},Q;Y_{2,Q}\right)+n\epsilon_{n}. \end{array}$$

Furthermore,

(24) $$\begin{array}{cc} nR_{1} & \leq I\left(M_{1};Y_{1}^{n},Y_{2}^{n},Y_{3}^{n}|M_{2}\right)+n\epsilon_{n}\\ & \overset{\left(a\right)}{=}\sum_{i=1}^{n}I\left(X_{i},M_{1};Y_{1,i},Y_{2,i},Y_{3,i}|U_{i},V_{i},X_{3,i},Y_{3}^{i-1}\right)+n\epsilon_{n}\\ & \leq \sum_{i=1}^{n}I\left(X_{i};Y_{1,i},Y_{2,i},Y_{3,i}|U_{i},V_{i},X_{3,i}\right)+n\epsilon_{n}\\ & \overset{\left(b\right)}{=}\sum_{i=1}^{n}I\left(X_{i};Y_{3,i}|U_{i},V_{i},X_{3,i}\right)+n\epsilon_{n}\\ & =nI\left(X_{Q};Y_{3,Q}|U_{Q},V_{Q},X_{3,Q},Q\right)+n\epsilon_{n}, \end{array}$$

where (a) holds by the definition of $\left(U_{i},V_{i}\right)$ and since $X_{3,i}$ is a function of $Y_{3}^{i-1}$; (b) holds due to the property of Type-I PDRBC, which has Markov chain $X_{i}-\left(U_{i},V_{i},X_{3,i},Y_{3,i}\right)-\left(Y_{1,i},Y_{2,i}\right)$.

Define $U=\left(Q,U_{Q}\right),V=\left(Q,V_{Q}\right),X=X_{Q},X_{3}=X_{3Q},Y_{1}=Y_{1Q},Y_{2}=Y_{2Q}$ and $Y_{3}=Y_{3Q}$. By the definition of $\left(U_{i},V_{i}\right)$ in (20), we have $V=\left(Q,V_{Q}\right)=\left(Q,Y_{1}^{Q-1},Y_{2}^{Q-1}\right)$ and $U=\left(Q,U_{Q}\right)=\left(Q,M_{2},Y_{1}^{Q-1},Y_{2}^{Q-1}\right)$, and thus:

$$H\left(V|U\right)=H\left(Q,Y_{1}^{Q-1},Y_{2}^{Q-1}|Q,M_{2},Y_{1}^{Q-1},Y_{2}^{Q-1}\right)=0,$$

and Markov chains $X_{3}-U-V$ and $V-(X_{3},U)-X$ hold, leading to pmf $p\left(u,v\right)p\left(x_{3}|u,v\right)p\left(x|x_{3},u\right)$ for this outer bound.

By Fano’s inequality [22], we know that $\epsilon_{n}$ tends to zero as n→∞. Combining (22)–(24), we obtain an outer bound as shown in Theorem 6.

### 7.2. Outer Bound for Type-II PDRBC with Feedback

Define:

(25) $$\begin{array}{c} U_{i}=\left(M_{2},Y_{1}^{i-1},Y_{2}^{i-1},Y_{3}^{i-1}\right). \end{array}$$

Introduce a time-sharing random variable *Q* that is uniformly distributed over [1:n] and independent of $\left(M_{1},M_{2},U^{n},X^{n},X_{3}^{n},Y_{1}^{n},Y_{2}^{n},Y_{3}^{n}\right)$.

By Fano’s inequality, we have:

(26) $$\begin{array}{ccc} nR_{1} & \leq & I\left(M_{1};Y_{1}^{n},Y_{2}^{n},Y_{3}^{n}|M_{2}\right)+n\epsilon_{n}\\ & \overset{\left(a\right)}{=} & \sum_{i=1}^{n}I\left(X_{i},M_{1};Y_{1,i},Y_{2,i},Y_{3,i}|U_{i},X_{3,i}\right)+n\epsilon_{n}\\ & \overset{\left(b\right)}{=} & \sum_{i=1}^{n}I\left(X_{i};Y_{1,i},Y_{2,i},Y_{3,i}|U_{i},X_{3,i}\right)+n\epsilon_{n}\\ & \overset{\left(c\right)}{=} & \sum_{i=1}^{n}I\left(X_{i};Y_{1,i}|U_{i},X_{3,i}\right)+n\epsilon_{n}\\ & = & nI\left(X_{Q};Y_{1,Q}|U_{Q},X_{3,Q},Q\right)+n\epsilon_{n}, \end{array}$$

where (a) holds because $X_{3,i}$ is a function of $Y_{3}^{i-1}$ and $X_{i}$ is a function of $\left(M_{1},M_{2},Y_{1}^{i-1},Y_{2}^{i-1},Y_{3}^{i-1}\right)$; (b) follows from the Markov chain $M_{1}-\left(U_{i},X_{i},X_{3,i}\right)-\left(Y_{1,i},Y_{2,i},Y_{3,i}\right)$; (c) holds due to the property of Type-II PDRBC, which has Markov chain $X_{i}-\left(U_{i},X_{3,i},Y_{1,i}\right)-\left(Y_{2,i},Y_{3,i}\right)$.

In addition,

(27) $$\begin{array}{ccc} R_{2} & \leq & I\left(M_{2};Y_{2}^{n}\right)+n\epsilon_{n}\\ & = & \sum_{i=1}^{n}I\left(M_{2};Y_{2,i}|Y_{2}^{i-1}\right)+n\epsilon_{n}\\ & \leq & \sum_{i=1}^{n}I\left(M_{2},Y_{1}^{i-1},Y_{2}^{i-1},Y_{3}^{i-1};Y_{2,i}\right)+n\epsilon_{n}\\ & = & \sum_{i=1}^{n}I\left(U_{i},X_{3,i};Y_{2,i}\right)+n\epsilon_{n}\\ & = & nI\left(U_{Q},X_{3,Q};Y_{2,Q}|Q\right)+n\epsilon_{n}\\ & \leq & nI\left(U_{Q},X_{3,Q},Q;Y_{2,Q}\right)+n\epsilon_{n}. \end{array}$$

Furthermore,

(28) $$\begin{array}{ccc} nR_{2} & \leq & I\left(M_{2};Y_{2}^{n},Y_{3}^{n}\right)+n\epsilon_{n}\\ & \overset{\left(a\right)}{=} & \sum_{i=1}^{n}I\left(M_{2};Y_{2,i},Y_{3,i}|Y_{2}^{i-1},Y_{3}^{i-1},X_{3,i}\right)+n\epsilon_{n}\\ & \leq & \sum_{i=1}^{n}I\left(M_{2},Y_{1}^{i-1},Y_{2}^{i-1},Y_{3}^{i-1};Y_{2,i},Y_{3,i}|X_{3,i}\right)+n\epsilon_{n}\\ & \overset{\left(b\right)}{=} & \sum_{i=1}^{n}I\left(U_{i};Y_{2,i},Y_{3,i}|X_{3,i}\right)+n\epsilon_{n}\\ & \overset{\left(c\right)}{=} & \sum_{i=1}^{n}I\left(U_{i};Y_{3,i}|X_{3,i}\right)+n\epsilon_{n}\\ & = & nI\left(U_{Q};Y_{3,Q}|X_{3,Q},Q\right)+n\epsilon_{n}\\ & \leq & nI\left(U_{Q},Q;Y_{3,Q}|X_{3,Q}\right)+n\epsilon_{n}, \end{array}$$

where (a) holds since $X_{3,i}$ is a function of $Y_{3}^{i-1}$; (b) holds by the definition of $U_{i}$; (c) holds due to the property of Type-II PDRBC, which has Markov chain $U_{i}-\left(X_{3,i},Y_{3,i}\right)-Y_{2,i}$. Here, the auxiliary random variable *Q* is not part of the channel variables and represents a convex combination of all rate points obtained in the absence of *Q*.

Define $U=\left(Q,U_{Q}\right),X=X_{Q},X_{3}=X_{3Q},Y_{1}=Y_{1Q},Y_{2}=Y_{2Q}$, and $Y_{3}=\left(Q,Y_{3Q}\right)$. By the definition of $U_{i}$ in (25), we have $U=\left(Q,U_{Q}\right)=\left(Q,M_{2},Y_{1}^{Q-1},Y_{2}^{Q-1},Y_{3}^{Q-1}\right)$, and since $X_{3}=X_{3,Q}$ is a function of $Y_{3}^{Q-1}$, we have Markov chain $X_{3}-U-X$, leading to pmf $p\left(u,x_{3}\right)p\left(x|u\right)$ for this outer bound.

By Fano’s inequality [22], $\epsilon_{n}$ tends to zero as n→∞. Combining (26)–(28), we obtain an outer bound the same as the inner bound of Theorem 1.

### 7.3. Outer Bound for Type-III PDRBC with Feedback

Define:

(29) $$\begin{array}{c} U_{i}=\left(M_{2},Y_{2}^{i-1},Y_{3}^{i-1}\right). \end{array}$$

Introduce a time-sharing random variable *Q* that is uniformly distributed over [1:n] and independent of $\left(M_{1},M_{2},X^{n},X_{3}^{n},Y_{1}^{n},Y_{2}^{n},Y_{3}^{n}\right)$.

By Fano’s inequality, we have:

(30) $$\begin{array}{cc} nR_{1} & \leq I\left(M_{1};Y_{1}^{n}\right)+n\epsilon_{n}\\ & \overset{\left(a\right)}{=}\sum_{i=1}^{n}I\left(X_{i},M_{1};Y_{1,i},Y_{2,i},Y_{3,i}|U_{i},X_{3,i}\right)+n\epsilon_{n}\\ & \overset{\left(b\right)}{=}\sum_{i=1}^{n}I\left(X_{i};Y_{1,i},Y_{2,i},Y_{3,i}|U_{i},X_{3,i}\right)+n\epsilon_{n}\\ & \overset{\left(c\right)}{=}\sum_{i=1}^{n}I\left(X_{i};Y_{1,i}|U_{i},X_{3,i}\right)+n\epsilon_{n}\\ & =nI\left(X_{Q};Y_{1,Q}|U_{Q},X_{3,Q},Q\right)+n\epsilon_{n}, \end{array}$$

where (a) holds since $X_{3,i}$ is a function of $Y_{3}^{i-1}$ and $X_{i}$ is a function of $\left(M_{1},M_{2},Y_{1}^{i-1},Y_{2}^{i-1},Y_{3}^{i-1}\right)$; (b) follows from the Markov chain $M_{1}-\left(U_{i},X_{i},X_{3,i}\right)-\left(Y_{1,i},Y_{2,i},Y_{3,i}\right)$; (c) holds due to the property of Type-III PDRBC, which has Markov chain $X_{i}-\left(U_{i},X_{3,i},Y_{1,i}\right)-\left(Y_{2,i},Y_{3,i}\right)$.

Furthermore,

(31) $$\begin{array}{ccc} nR_{2} & \leq & I\left(M_{2};Y_{2}^{n},Y_{3}^{n}\right)+n\epsilon_{n}\\ & = & I\left(M_{2};Y_{2,i},Y_{3,i}|Y_{2}^{i-1},Y_{3}^{i-1}\right)+n\epsilon_{n}\\ & = & I\left(M_{2};Y_{2,i},Y_{3,i}|Y_{2}^{i-1},Y_{3}^{i-1},X_{3,i}\right)+n\epsilon_{n}\\ & \overset{\left(a\right)}{\leq } & I\left(U_{i};Y_{2,i},Y_{3,i}|X_{3,i}\right)+n\epsilon_{n}\\ & \overset{\left(b\right)}{=} & I\left(U_{i};Y_{2,i}|X_{3,i}\right)+n\epsilon_{n}\\ & \leq & I\left(U_{Q},Q;Y_{2,Q}|X_{3,Q}\right)+n\epsilon_{n}, \end{array}$$

where (a) holds by the definition of $U_{i}$; (b) holds due to the property of Type-III PDRBC, which has Markov chain $U_{i}-\left(X_{3,i},Y_{2,i}\right)-Y_{3,i}$. Here, the auxiliary random variable *Q* is not part of the channel variables and represents a convex combination of all rate points obtained in the absence of *Q*.

Define $U=\left(Q,U_{Q}\right),X=X_{Q},X_{3}=X_{3Q},Y_{1}=Y_{1Q},Y_{2}=Y_{2Q}$, and $Y_{3}=\left(Q,Y_{3Q}\right)$. By the definition of $U_{i}$ in (29), we have $U=\left(Q,U_{Q}\right)=\left(Q,M_{2},Y_{2}^{Q-1},Y_{3}^{Q-1}\right)$, and since $X_{3}=X_{3,Q}$ is a function of $Y_{3}^{Q-1}$, we have Markov chain $X_{3}-U-X$, leading to pmf $p\left(u,x_{3}\right)p\left(x|u\right)$ for this outer bound.

By Fano’s inequality [22], $\epsilon_{n}$ tends to zero as n→∞. Combing (31) and (30), we obtain the outer bound as below.

$$\begin{array}{ccc} R_{2} & \leq & I\left(U;Y_{2}|X_{3}\right)=\sum_{x_{3}\in X_{3}}I\left(U;Y_{2}|X_{3}=x_{3}\right),\\ R_{1} & \leq & I\left(X;Y_{1}|U,X_{3}\right)=\sum_{x_{3}\in X_{3}}I\left(X;Y_{1}|U,X_{3}=x_{3}\right), \end{array}$$

for some pmf $p\left(u,x_{3}\right)p\left(x|u\right)$. Note that $I(U;Y_{2}|X_{3}=x_{3})$ and $I(X;Y_{1}|U,X_{3}=x_{3})$ both are linear functions of p(x,u), and since $X_{3}$ is constant, the boundary points on the outer bound:

$$\sum_{x_{3}\in X_{3}}\left(I\left(U;Y_{2}|X_{3}=x_{3}\right)+I\left(X;Y_{1}|U,X_{3}=x_{3}\right)\right)$$

are maximized at an extreme point. Thus, the corresponding outer bound can be characterized as:

$$\begin{array}{ccc} R_{2} & \leq & I\left(U;Y_{2}|X_{3}\right)=I\left(U;Y_{2}|X_{3}=x_{3}\right),\\ R_{1} & \leq & I\left(X;Y_{1}|U,X_{3}\right)=I\left(X;Y_{1}|U,X_{3}=x_{3}\right), \end{array}$$

for some value $x_{3}\in X_{3}$ and pmf p(x,u), which completes the converse.

## 8. Proof of Theorem 2

A rigorous proof that the inner bounds in Equation (1) and Theorem 1 both hold for the Gaussian PDRBC is omitted for brevity. In the following subsections, we will first prove the achievability of rate region (8) and (9) and then show that these inner bounds are tight.

### 8.1. Capacity Region on Type-II Gaussian PDRBC

(1) Proof of the achievability:
  Let:
  $$\begin{array}{c} U=\rho_{1}X_{3}+W_{1},\;X=\rho_{2}U+W_{2}, \end{array}$$
  where $\left(X_{3},W_{1},W_{2}\right)$ are independent of each other and $X_{3}∼N\left(0,P_{r}\right)$, $\rho_{2}W_{1}∼N\left(0,\left(\beta -\alpha \right)P\right)$, $\rho_{2}U∼N\left(0,\bar{\alpha }P\right)$, and $W_{2}∼N\left(0,\alpha P\right)$, with $0\leq \rho_{1},\rho_{2},\alpha ,\beta \leq 1$, and β≥α. With the choice above, we obtain:
  $$\begin{array}{ccc} R_{2} & \leq & I\left(U,X_{3};Y_{2}\right)=C\left(\frac{\bar{\alpha }P+P_{r}}{\alpha P+\sigma_{2}^{2}}\right),\\ R_{2} & \leq & I\left(U;Y_{3}|X_{3}\right)=C\left(\frac{(\beta -\alpha )P}{\alpha P+\sigma_{3}^{2}}\right),\\ R_{1} & \leq & I\left(X;Y_{1}|U,X_{3}\right)=C\left(\frac{\alpha P}{\sigma_{1}^{2}}\right), \end{array}$$
  where $0\leq \rho_{1},\rho_{2},\alpha ,\beta \leq 1$, and β≥α.
(2) Proof of the converse:
  Consider:
  $$\begin{array}{c} I\left(U;Y_{3}|X_{3}\right)=h\left(Y_{3}|X_{3}\right)-h\left(Y_{3}|X_{3},U\right), \end{array}$$
  since:
  $$\begin{array}{cc} \frac{1}{2}\log\left(2\pi e\sigma_{3}^{2}\right) & =h\left(Z_{3}\right)\leq h\left(Y_{3}|X_{3}\right)\leq h\left(X+Z_{3}\right)\\ & \leq \frac{1}{2}\log\left(2\pi e(P+\sigma_{3}^{2})\right), \end{array}$$
  there must exist a β∈[0,1] such that:
  $$h\left(Y_{3}|X_{3}\right)=\frac{1}{2}\log\left(2\pi e(\beta P+\sigma_{3}^{2})\right).$$
  Similarly, since:
  $$\begin{array}{cc} \frac{1}{2}\log\left(2\pi e\sigma_{3}^{2}\right) & =h\left(Z_{3}\right)\leq h\left(Y_{3}|U,X_{3}\right)\leq h\left(Y_{3}|X_{3}\right)\\ & =\frac{1}{2}\log\left(2\pi e(\beta P+\sigma_{3}^{2})\right), \end{array}$$
  there must exist an α∈[0,β] such that:
  (32) $$\begin{array}{c} h\left(Y_{3}|U,X_{3}\right)=\frac{1}{2}\log\left(2\pi e(\alpha P+\sigma_{3}^{2})\right). \end{array}$$
  Thus,
  $$R_{2}\leq I\left(U;Y_{3}|X_{3}\right)=C\left(\frac{(\beta -\alpha )P}{\alpha P+\sigma_{3}^{2}}\right).$$
  Next, consider:
  $$\begin{array}{c} I\left(U,X_{3};Y_{2}\right)=h\left(Y_{2}\right)-h\left(Y_{2}|U,X_{3}\right)\\ \;\leq \frac{1}{2}\log\left(2\pi e(P+P_{r}+\sigma_{2}^{2})\right)-h\left(Y_{2}|U,X_{3}\right). \end{array}$$
  By (32) and the conditional EPI in [22], we have:
  $$\begin{array}{ccc} h(Y_{2}|U,X_{3}) & = & h(Y_{3}+{\tilde{Z}}_{b}|U,X_{3})\\ & \geq & \frac{1}{2}\log\left(2^{2h(Y_{3}|U,X_{3})}+2^{2h({\tilde{Z}}_{b}|U,X_{3})}\right)\\ & = & \frac{1}{2}\log\left(2\pi e\left(\alpha P+\sigma_{3}^{2}\right)+2\pi e\left(\sigma_{2}^{2}-\sigma_{3}^{2}\right)\right)\\ & = & \frac{1}{2}\log\left(2\pi e(\alpha P+\sigma_{2}^{2})\right). \end{array}$$
  Thus,
  $$R_{2}\leq I\left(U,X_{3};Y_{2}\right)\leq C\left(\frac{\bar{\alpha }P+P_{r}}{\alpha P+\sigma_{2}^{2}}\right).$$
  Now, consider:
  $$\begin{array}{cc} I(X;Y_{1}|U,X_{3}) & =h\left(Y_{1}|U,X_{3}\right)-h\left(Z_{1}\right)\\ & =h\left(Y_{1}|U,X_{3}\right)-\frac{1}{2}\log\left(2\pi \sigma_{1}^{2}\right), \end{array}$$
  since:
  (33) $$\begin{array}{cc} h(Y_{3}|U,X_{3}) & =h(Y_{1}+{\hat{Z}}_{b}|U,X_{3})\\ & \geq \frac{1}{2}\log\left(2^{2h(Y_{1}|U,X_{3})}+2^{2h({\hat{Z}}_{b}|U,X_{3})}\right)\\ & =\frac{1}{2}\log\left(2^{2h(Y_{1}|U,X_{3})}+2\pi e\left(\sigma_{3}^{2}-\sigma_{1}^{2}\right)\right). \end{array}$$
  Combining (32) with (33), we obtain:
  $$2\pi e\left(\alpha P+\sigma_{3}^{2}\right)\geq 2^{2h(Y_{1}|U,X_{3})}+2\pi e\left(\sigma_{3}^{2}-\sigma_{1}^{2}\right).$$
  Thus,
  $$h\left(Y_{1}|U,X_{3}\right)\leq \frac{1}{2}\log\left(2\pi e(\alpha P+\sigma_{1}^{2})\right),$$
  which implies:
  $$R_{1}\leq I\left(X;Y_{1}|U,X_{3}\right)\leq C\left(\frac{\alpha P}{\sigma_{1}^{2}}\right).$$
  This completes the proof of the converse.

### 8.2. Capacity Region on Type-III Gaussian PDRBC

(1) Proof of the achievability:
  The achievability follows by the traditional superposition coding and by shutting down the relay, i.e., set:
  X=U+V,
  where $U∼N(0,\bar{\alpha }P)$ and V∼N(0,αP) are independent of each other. With this choice, it is easy to obtain the rate region in (8).
(2) Proof of the converse
  Consider:
  $$\begin{array}{cc} I(U;Y_{2}|X_{3}) & =h\left(Y_{2}|X_{3}\right)-h\left(Y_{2}|U,X_{3}\right)\\ & \leq \frac{1}{2}\log\left(2\pi e(P+P_{r}+\sigma_{2}^{2})\right)-h\left(Y_{2}|U,X_{3}\right). \end{array}$$
  Since:
  $$\begin{array}{ccc} \frac{1}{2}\log\left(2\pi e\sigma_{2}^{2}\right) & = & h\left(Z_{2}\right)\leq h\left(Y_{2}|U,X_{3}\right)\leq h\left(Y_{2}|X_{3}\right)\\ & \leq & \frac{1}{2}\log\left(2\pi e(P+\sigma_{2}^{2})\right), \end{array}$$
  there must exist an α∈[0,1] such that:
  (34) $$\begin{array}{c} h\left(Y_{2}|U,X_{3}\right)=\frac{1}{2}\log\left(2\pi e(\alpha P+\sigma_{2}^{2})\right). \end{array}$$
  Thus:
  $$R_{2}\leq I\left(U;Y_{2}|X_{3}\right)\leq C\left(\frac{\bar{\alpha }P}{\alpha P+\sigma_{2}^{2}}\right).$$
  Next, consider:
  $$\begin{array}{cc} I(X;Y_{1}|U,X_{3}) & =h\left(Y_{1}|U,X_{3}\right)-h\left(Y_{1}|X,X_{3}\right)\\ & =h\left(Y_{1}|U,X_{3}\right)-\frac{1}{2}\log\left(2\pi e\sigma_{1}^{2}\right). \end{array}$$
  Using the conditional EPI, we obtain:
  (35) $$\begin{array}{cc} h(Y_{2}|U,X_{3}) & =h(Y_{1}+{\hat{Z}}_{c}|U,X_{3}).\\ & \geq \frac{1}{2}\log\left(2^{2h(Y_{1}|U,X_{3})}+2^{2h({\hat{Z}}_{c}|U,X_{3})}\right)\\ & =\frac{1}{2}\log\left(2^{2h(Y_{1}|U,X_{3})}+2\pi e\left(\sigma_{2}^{2}-\sigma_{1}^{2}\right)\right). \end{array}$$
  Combining (34) and (35), we have:
  $$2\pi e\left(\alpha P+\sigma_{2}^{2}\right)\geq 2^{2h(Y_{1}|U,X_{3})}+2\pi e\left(\sigma_{2}^{2}-\sigma_{1}^{2}\right),$$
  which implies:
  $$h\left(Y_{1}|U,X_{3}\right)\leq \frac{1}{2}\log\left(2\pi e(\alpha P+\sigma_{1}^{2})\right),$$
  and hence:
  $$R_{1}\leq I\left(X;Y_{1}|U,X_{3}\right)\leq C\left(\frac{\alpha P}{\sigma_{1}^{2}}\right).$$
  This completes the proof of the converse.

## 9. Conclusions

In this paper, we consider three types of PDRBCs that vary with different degradation orders among the relay and receiver’s observed signals. For the first type of DM-PDRBC, a new outer bound is established having the same rate expression as an existing inner bound, with only a slight difference in the input distributions; for the second type of Gaussian PDRBC, the capacity region is established; for the third type of PDRBC, the capacity regions are established both for DM and the Gaussian case. Furthermore, we propose a new scheme for the general RBC with feedback from the relay node to the transmitter. It is shown that our scheme can strictly enlarge Behboodi and Piantanida’s rate region, which achieves the capacity region of the second type of DM-PDRBC. Moreover, we show that capacity regions of the second and third types of PDRBCs are exactly the same as those without feedback, which means that feedback cannot enlarge capacity regions for these types of RBCs.
